# Neuro-functional modeling of near-death experiences in contexts of altered states of consciousness

**DOI:** 10.3389/fpsyg.2022.846159

**Published:** 2023-01-18

**Authors:** Raymond Romand, Günter Ehret

**Affiliations:** ^1^Faculty of Medicine, University of Strasbourg, Strasbourg, France; ^2^Institute of Neurobiology, University of Ulm, Ulm, Germany

**Keywords:** brain death, hallucinations, hypoxia, ischemic stress, NDEs/OBEs/G-LOCs, neuro-functional models, out-of-body experience

## Abstract

Near-death experiences (NDEs) including out-of-body experiences (OBEs) have been fascinating phenomena of perception both for affected persons and for communities in science and medicine. Modern progress in the recording of changing brain functions during the time between clinical death and brain death opened the perspective to address and understand the generation of NDEs in brain states of altered consciousness. Changes of consciousness can experimentally be induced in well-controlled clinical or laboratory settings. Reports of the persons having experienced the changes can inform about the similarity of the experiences with those from original NDEs. Thus, we collected neuro-functional models of NDEs including OBEs with experimental backgrounds of drug consumption, epilepsy, brain stimulation, and ischemic stress, and included so far largely unappreciated data from fighter pilot tests under gravitational stress generating cephalic nervous system ischemia. Since we found a large overlap of NDE themes or topics from original NDE reports with those from neuro-functional NDE models, we can state that, collectively, the models offer scientifically appropriate causal explanations for the occurrence of NDEs. The generation of OBEs, one of the NDE themes, can be localized in the temporo-parietal junction (TPJ) of the brain, a multimodal association area. The evaluated literature suggests that NDEs may emerge as hallucination-like phenomena from a brain in altered states of consciousness (ASCs).

## Introduction

The focus of our present review is the etiology of personal experiences which have fascinated, for a long time, both scientific, medical and esoteric communities, the near-death experiences (NDEs). Although reports about NDEs are known from many cultural backgrounds and for many centuries (Zaleski, 1987; Schlieter, 2018; Shushan, 2018; Peinkhofer et al., 2021) this term was introduced to a broad English speaking public by Moody (1975) reviewing reports of patients from the USA having survived life-threatening situations (e.g., car accidents, drowning, cardiac arrest). An example of an NDE narrative recorded after a car accident can be found in Supplementary material 1. Very often the content of the reports was emotionally touching when details were given about seeing dead relatives, past experiences, traveling the cosmos, meeting strange entities like divine figures, or visualizing a brilliant entity sometimes called the light. Often associated with NDEs were out-of-body experiences (OBEs) described as perceiving the world from a location outside of the own physical body (Blackmore, 1992, 2017; Blanke and Dieguez, 2009; Shushan, 2018).

Here, we evaluate scientific evidence about the generation of NDEs including OBEs and provide a solid perspective for studying the etiologies of these experiences in the context of brain-based altered states of consciousness (ASCs). In the broad context of neuropsychology, ASCs are approached as deviations from the states of wakefulness and/or awareness which are the two main conditions allowing consciousness to be generated. Wakefulness refers to that daily state in the circadian rhythm of sleeping and waking when a person can interact with the world and engage in coherent activities (Hobson, 2009); awareness refers to the subjective feeling of actually being aware of something (e.g., Sattin et al., 2021). This subjective awareness of something has also been termed *phenomenal consciousness* (Block, 2005). Further terms of consciousness concern, for example, *access consciousness*, the memory- and cognition-based action planning and execution (Block, 2005) and self-consciousness or self-awareness (e.g., Vogeley and Fink, 2003; Morin, 2006; Singer, 2019). Phenomenal consciousness and access consciousness can be characterized by different neural correlates of consciousness in activity recordings of the brain (e.g., Ehret and Romand, 2022). Opposite to wakefulness and awareness, loss of consciousness (LOC) corresponds to the absence of subjective experience and refers to a state in which an individual lacks the normal awareness of the self and the surrounding environment (e.g., Laureys, 2005; Bonhomme et al., 2019). ASCs are found in the broad range between the fully awake awareness and complete LOC as observed in coma (e.g., Kondziella et al., 2020).

## How have near-death experiences been defined?

Three definitions and a set of components regarded as necessary elements of NDEs shall be presented. They somehow reflect the historical course of scientific thinking about NDEs. The first NDE definition was proposed by Moody (1975), a psychiatrist:

“…*any conscious perceptual experience which takes place during a ‘near encounter,’ an event in which a person could very easily die or be killed (and may even be so close to be believed or pronounced clinically dead) but none the less survives, and continues physical life.”*

It is interesting to note that, initially, Moody put forward the idea that NDEs may be based on “*conscious perceptual experience*,” although the persons were unconscious and often clinically dead during the time of their experience. Later, Greyson, a dedicated psychiatrist in the field of NDE research, introduced the term “*transcendental*,” giving a perspective with relation to the paranormal (Greyson, 1983):

“*NDEs are profound psychological events with transcendental and mystical elements, typically occurring to individuals close to death or in situations of intense physical or emotional danger.*”

Charland-Verville et al. (2020) from a coma studying group, proposed a more pragmatic and topical definition:

“*NDEs as a set of mental events including highly emotional, self-related, mystical and spiritual aspects occurring in an altered state of consciousness classically in the context of a life-threatening condition.”*

Recently, a group of researchers from the USA and UK (Parnia et al., 2022) proposed a set of six components that should occur in reports of experiences in relation to death in order to be called NDE, which they renamed RED (recalled experience of death or authentic NDE):

*“(1) a relation with death, (2) a sense of transcendence, (3) ineffability, (4) positive transformative effects (related to meaning and purpose to life), and a (5) severity of illness that leads to loss of consciousness (LOC), together with the (6) absence of features of other coma-related experiences (such as conventional dreams, delirium, and delusions, in the intensive care unit (ICU) or elsewhere).”*

Important differences between these definitions concern the inclusion of transcendental elements (Greyson, 1983; Parnia et al., 2022), and the implication of consciousness (Moody, 1975) or LOC (Parnia et al., 2022) or ASCs (Charland-Verville et al., 2020). Thus, these definitions reflect basic differences in how to approach the understanding of the phenomena of NDEs (including OBEs). The NDE definition by Charland-Verville et al. (2020) and the consideration that some situations of NDEs may refer to disconnected consciousness as, for example, observed in dreaming during rapid-eye-movement sleep (Martial et al., 2020a) do not only acknowledge a psychological basis of NDEs (Lange et al., 2004) but also, and primarily, a neurophysiological basis becoming noticeable in ASCs. This NDE definition opens the door to the understanding of NDEs *via* the knowledge of the neurophysiological bases of the generation of wakefulness and awareness and of changes in these basic attributes of consciousness (e.g., Sperry, 1968; Crick and Koch, 1990; Edelman, 1992; Baars, 1993; Aru et al., 2012; Damasio, 2012; Dehaene, 2014; Tononi et al., 2016; Gazzaniga, 2018; Ehret and Romand, 2022). The definition of an authentic NDE or RED by listing necessary presence and absence of components in the NDE reports has been criticized (Evrard et al., 2022) as “*a very risky strategy, which truncates the NDE phenomenon to avoid confronting what the last decades of research have revealed.”*

Given the obvious differences in NDE definition, there seems agreement about consciousness of some kind (LOC, ASCs) being involved in the generation of NDEs. Further, it is fact that many topics or themes in NDE narratives can be found in reports characterizing experiences induced by modified brain functions which not necessarily imply LOC (see section below on “Explanatory models of near-death experiences including out-of-body experiences: Neuro-functional approaches”). We will name these experiences NDE-like. The relationship between NDEs, NDE-like phenomena and consciousness has been discussed in the context of various states and conditions of consciousness (Martial et al., 2020a). Here, we will open the perspective to scientifically address and validate NDEs in the context of brain functions. Their relationship to consciousness has been a major subject of studies in the neurosciences for a long time (e.g., Ehret and Romand, 2022). Such a neuro-functional approach may offer valuable ideas for psychiatric and psychologic learning- and/or medication-based treatment of persons who suffer from their experiences.

## How have near-death experiences been recorded and analyzed?

The content of an NDE report is the narrative describing the subjective experience of a person having survived life-threatening conditions. The reports have usually been collected by family members or health professionals, hours, days, weeks, or more up to several years after the NDE experiencer (NDEr) had returned to consciousness from an unconscious phase which could have lasted minutes, hours, days, weeks or several months or years as a result of a life-threatening event. Thus, these reports and the studies based on the reports are naturally retrospective, uncontrolled, unblinded, and possibly heavily biased by the health, religion, culture, social relations, lifestyle, etc., of the NDEr at the time of reporting, and the interview style (neutral, suggestive, demanding) of the person asking for the report (Marsh, 2010). More recent reports of NDErs have also been obtained in more standardized ways or in more controlled surroundings after cardiac arrest resuscitation in medical settings (Parnia et al., 2001, 2014; van Lommel et al., 2001; Greyson et al., 2006). Therefore, the latter reports shall be taken as the most informative ones, when relationships between the various themes in the reports and their possible psychological and physiological backgrounds are in the focus of research.

In general, about 9–20% of the people having survived life-threatening conditions such as clinical death reported NDEs (Greyson, 1998; Schwaninger et al., 2002; Kondziella, 2020). Content or themes of the reports have been listed in many studies (Moody, 1975; Rawlings, 1978; Ring, 1980; Sabom, 1983; Grey, 1985; Kübler-Ross, 1991; Morse and Perry, 1991; Fenwick and Fenwick, 1997). NDErs reported about (among other things) seeing a bright warm light, sensing comfort and peace, wonderful locations, sweet feelings, extreme speed of thought and imagination, a rapid view of the whole course of past life, OBEs, an elastic bond between body and soul, a dark tunnel, meeting spiritual beings and others, entering another world, reaching an alternate value system.

Lists of major themes occurring in NDE reports have been derived from standardized questionnaires used in interviews of large numbers of potential NDErs (e.g., Greyson, 1983; Agrillo, 2011; Parnia, 2014). Such approved lists can be useful as a statistically meaningful basis for:

(1) identifying potential NDErs,
(2) separating NDE-related from other stories of a potential NDEr,
(3) ranking themes according to their prevalence and relative frequency of occurrence in order to evaluate possible psychological and neurological relationships *via*
(4) testing for differences in experiences between age/sex/social/cultural/etc., groups,
(5) comparing themes from NDE reports with themes having been reported after awakening from unconsciousness caused by non-life-threatening events (e.g., sleep, anesthesia, drug consumption, epileptic seizures) in order to identify relevant mimics and scientific alternatives for studying the causation of NDE themes,
(6) relating themes to likely neural substrates known to be involved in generating certain perceptual experiences.

More recently, qualitative and quantitative text analyses have also been used to characterize the content-related structure and/or the frequency of occurrence of certain words in NDE reports. Sequences of themes such as OBEs, experiencing a tunnel, seeing a bright light, and feeling of peace have been found (Martial et al., 2017), time-bounded and transversal themes (Cassol et al., 2018), and clusters of themes with a high incidence of esoteric ones (Lange et al., 2015) have been identified, as well as a prevalence of the usage of the words “light,” “well,” “see,” “body” (Charland-Verville et al., 2020).

The focus of our present analysis will be on the aspect (3) in combination with (5) and (6) of the above list, which we will develop in combining rather unvalued old data from NDE-like contexts with more recent evidence about brain activity under physiological stress such as near death. In this context, we rely on the widely accepted and used NDE scale of Greyson (1983). The scale was initially constructed by selecting 80 items from existing NDE reports and subsequently reducing the number of features to a finally validated 16-item questionnaire. Each of the 16 questions (see Supplementary material 2) covered a specific NDE theme and required an answer by one of three given possibilities of different weights. The weighted answers led to a scale with statistics according to which a total score of 7 or higher qualified a person as an NDEr. Persons whose experience did not sufficiently meet the 16 NDE themes covered, or otherwise did not reach the accepted cut-off score would not be considered as an NDEr. Thus, the scale served according to aspects (1) and (2) of the above list and, according to aspect (3), presenting 16 themes (among them 4 paranormal and 4 transcendental themes) for which could be searched in reports of persons awakening from unconsciousness or altered consciousness of various genesis. Recently, this scale has been reassessed, now containing 20 items grouped in five clusters (Martial et al., 2020b).

Although the perceptions/feelings of the great majority of NDEs have been described as positive or neutral (these are the ones usually occurring in the lists of NDE themes, see Supplementary material 2 with the scale of Greyson, 1983), negative themes could also occur such as very frightening and alarming contexts, feeling very lonely or non-being, being in a hellish environment and terrified by impending judgment, and torment (Greyson and Bush, 1992; Bush, 2002; Bush and Greyson, 2014). In a sample of 123 NDE reports, Cassol et al. (2019) found 17 (14%) with such negative perceptions. Some NDErs were left with long-term cognitive impairments and psychological sequelae such as post-traumatic stress disorder (Parnia et al., 2007). Such negative themes and their life consequences have not been acknowledged as REDs in the recent review of Parnia et al. (2022).

## Brain function in between clinical death and brain death

Before we approach brain-based functional models of NDEs or NDE-like experiences, it is helpful to be clear with the distinction between clinical death and brain death. Today, clinical death covers what people historically termed as death. Clinical death is diagnosed when a subject has no heart function, has no more reflexes, does not breathe, and does not respond to repeated well-defined clinical tests, e.g., of muscular activation (e.g., Howard and Leaman, 2001). With improved medical technology of cardiopulmonary resuscitation, it is possible now to overcome clinical death, i.e., to restore cardiac function and breathing after an arrest of 10–15 min or longer. Modern techniques even allowed the inducing of clinical death or artificial coma in order to save the lives of severely injured trauma victims while trying to repair their injuries (e.g., Young et al., 2002). Therefore, one has to understand that clinical death is not death, but brain death implies the termination of a human’s life (Wijdicks, 2015).

The concept of brain death first emerged in the 1950s driven by progress in critical care medicine such as cardiopulmonary resuscitation and the development of mechanical ventilation (Field et al., 2010; Wahlster et al., 2015). The diagnostic criteria for assessing brain death in addition to clinical death may differ among countries, however, all include measurements of possible brain activity, at least *via* EEGs (Wahlster et al., 2015; Kondziella, 2020). Thus, with technological progress, the concept of death has evolved from an ancient cardiorespiratory-centered diagnosis to a neuro-centered diagnosis (Bernat, 2004; Laureys, 2005). No recovery from brain death has ever been reported in patients fulfilling the criteria of brain death according to the first clinical/neurological definition of brain death nearly 50 years ago (Laureys et al., 2009).

What does this distinction between clinical death and brain death mean for the causal understanding of NDEs? With normal body temperature and without medical intervention, brain death occurs 4 to six 6 min after clinical death (Lind et al., 1975). Normally within about 10 s after circulatory arrest unconsciousness starts (Rossen et al., 1943). After about 5 min, substantial injury of the brain cells may begin (Neumar et al., 2008). It has been shown that some neurons of the brain are more resistant to the effects of anoxic injury than others (Vaagenes et al., 1996; Kirino, 2000; Taoufik and Probert, 2008). Among them are neurons of the hippocampus and amygdala (Kirino, 2000; Kawahara et al., 2004), key brain structures in cognitive and emotional processes (Janak and Tye, 2015). The underlying physiological differences between hypoxia-sensitive and less sensitive neurons are not yet clear. What is clear, however, is the fact that neurons of the brain do not become irreversibly damaged or dead immediately after assessment of clinical death. There is a time window of roughly 5 min of progression toward irreversible damage ending with brain death. As Parnia et al. (2014) stated: “…cardiac arrest [i.e., ‘clinical death,’ our addition] is the most physiologically appropriate and accessible biological state for research into the actual experience of death.” As detailed below, data on physiological measurements of brain activity after cardiac arrest are actually available. Such data may be related to NDE reports of persons having been rescued from brain death in order to get informed about the individual experience accompanied by the changes in the function of a brain in extreme metabolic stress.

What can we learn from changes in the EEG in the roughly 5 min of transition from cardiac arrest to beginning brain injury? New technologies of cortical activity reading used in intensive medical care units led to the Bispectral Index (BIS) in the analysis of frontal cortical EEG signals (Sigl and Chamoun, 1994). The digitized and processed EEG could produce a BIS score between 0 (flat EEG) and 100 (EEG of a fully awake and conscious adult). Such EEG scores have been applied to dying patients at or near the moment of death. The patients were sedated by some medication, mostly morphine or midazolam. Part of the studied patients, who had not been diagnosed before as being brain-dead, revealed startling end-of-life brain activity (Chawla et al., 2009, 2017). Cardiac arrests were followed by a decline of the BIS score but then followed by a sudden transient BIS score increase to levels approaching those of normal consciousness. This transiently high EEG activity had a maximum duration of a few minutes and then declined to a low level. The BIS should be used with caution because electromagnetic and electrostatic artifacts may lead to misinterpretations of signals giving an incorrect state of the brain (Dahaba, 2005; Lee et al., 2019), in some cases due to myogenic contaminations (Yilmaz et al., 2014). If care has been taken that the EEG recordings and BIS assessment have not been contaminated by artifacts, these observations suggest that, as the brain reaches a critical level of hypoxia, the Na-K resting potential is lost rather simultaneously by large numbers of neurons (Chawla et al., 2009, 2017). This loss causes a cascade of electrical activity becoming obvious in a high EEG current which rapidly dissipates. With the background of these measurements, Chawla et al. (2009, 2017) speculated that patients successfully revived after cardiac arrest may recall the images and memories triggered by this neural activity cascade. They offered this as a potential explanation for the clarity in which many patients report about NDEs when successfully revived from a near-death event.

More recently, Dreier et al. (2018; see also Dreier, 2011; Carlson et al., 2019) were able to record intracortical activity of dying patients due to cardiac arrest (clinical death). They reported a drastic drop in the brain’s arterial pressure just after cardiac arrest. At the same time, a sharp decline in neural activity appeared throughout the brain. This non-spreading depression was associated with neuronal hyperpolarization. This activity decrease developed during the steep fall of brain oxygenation and may correspond to cellular energy-saving necessary to preserve brain cell integrity. It lasted 2 to 3 min. With a latency of up to 4–5 min, a phase of activity of chained neurons followed spreading toward neighboring neurons throughout the brain for about 10 min (spreading depolarization). The onset of spreading depolarization marked the start of toxic processes to the neurons which, however, could still be reversed by adequate resuscitation procedures. Finally, there was a transition from spreading depolarization to a negative ultraslow potential signaling the dying and death of neurons.

This recorded sequence of changes in neuronal activity—non-spreading depression, spreading depolarization, negative ultraslow potential–is a characteristic response of brain areas to the loss of energy supply not only after cardiac arrest but also after ischemia of other geneses, e.g., traumatic brain injury or hemorrhage (Sugimoto et al., 2018; Hartings et al., 2020; Dreier et al., 2022; Lemale et al., 2022). Again, after cardiac arrest and other causes of loss of energy supply a phase of high brain activity was characteristic before the brain died. Hence, if early enough rescued from brain death, the human subjects might have reported subjective experiences equivalent to NDEs caused by the highly active brain.

Recent recordings of EEG patterns from an 87-year-old patient having been hospitalized after traumatic subdural hematoma and dying because of cardiac arrest specified the brain activity of a dying person with regard to brain-wave occurrence (Vicente et al., 2022). Brain waves were analyzed with regard to the temporal occurrence and spatial cortical distribution of the various waves, especially gamma-waves. Gamma-waves are associated with brain activity during states, for example, of sensory perception, memory retrieval, waking, dreaming, meditation (Llinás and Paré, 1991; Steriade et al., 1996; Llinás et al., 1998; Fries, 2009; Bosman et al., 2014) and aware perception and conscious action planning (e.g., Ehret and Romand, 2022). The data showed coupling of brain waves and long-range communication *via* gamma-waves in a well-coordinated way between sensory, motor, and association areas of the brain. Such a gamma-wave occurrence is typical for perception and associative use of memory content in a conscious person (Canolty et al., 2006; Jensen and Colgin, 2007; Dehaene and Changeux, 2011; Li et al., 2014), even if the perception was illusionary (Faramarzi et al., 2021). Therefore, brain activity of a dying person due to cardiac arrest does not appear disorganized or erratic. Instead, it shows phases of coordinated coupling and oscillatory activity resembling memory recall and sensory and sensory-motor associations as if the person experienced NDE themes in a state of altered consciousness (Chawla et al., 2009, 2017; Thonnard et al., 2013; Palmieri et al., 2014). Interestingly, a transient surge of well-synchronized gamma-waves (also phase-coupled with theta- and alpha-waves) has been recorded from the rat brain after cardiac arrest (Borjigin et al., 2013). Hence, data from both humans and rats suggest that after cardiac arrest the brain of a subjectively non-conscious subject may function in a mode typical for awareness and conscious behaving. On the other hand, recordings from the rat brain during induction of and resuscitation from anoxic stress suggest that NDEs may be generated rather in the late phase of recovery (before becoming conscious again) than in the initial LOC phase (Schramm et al., 2020). Further observations can test the hypothesis that systematic changes of brain waves, especially gamma-waves, may be associated with NDE generation in a brain becoming stressed by and/or recover from hypoxia and energy loss.

### In summary

It seems to be possible to obtain relevant prospective data about brain activation during the time of NDE generation in a highly controlled clinical setting. Necessary conditions would be cardiac arrest, LOC, and extensive measurements of the course of brain activity in relation to other physiological parameters. After the person had regained consciousness, possible NDE reports could be evaluated (e.g., according to Greyson, 1983, or other criteria) and related to neural and physiological measurements during the unconscious phase. The expected brain activation might show patterns of EEG waves resembling those of conscious perception and action planning.

## Explanatory models of near-death experiences including out-of-body experiences: Neuro-functional approaches

The just described clinical scenario of mimicking clinical death with the progression toward brain death, however, without allowing permanent brain impairment after resuscitation would be a very complex and expensive way, sensibly attached to an otherwise necessary major surgery, to possibly obtain NDE reports with scientific scrutiny. Purely experimental approaches in healthy humans aiming to measure physiological and behavioral consequences of a temporal blocking of the main blood supply to the brain (Rossen et al., 1943; see also Smith et al., 2011) would be judged unethical and disapproved today. Clearly, there is demand for other ways of scientific validation of NDEs as neuro-functional phenomena. The important common goal of neuro-functional approaches to the causal understanding of NDE features consists of the reproduction of NDE themes listed in the mentioned NDE literature together with an approach-specific measure of related brain activation or activation changes.

## Approaches *via* modification of brain functions by

### Drugs

The effects of many chemicals in altering consciousness can be predicted by their structure and their binding properties to receptors of various neurotransmitters or neuromodulators in the brain (e.g., Perrine, 1996; Nichols, 2016). A comprehensive review and analysis (Martial et al., 2019) of 625 NDE-like narratives referring to 165 different substances in 10 drug classes provided very useful insight. The analysis uncovered remarkable similarities between the psychological effects of certain drugs—first of all ketamine. Ketamine is a drug widely used for anesthetic medications (Pal et al., 2015; Bonhomme et al., 2016). It blocks cellular receptors in the brain for the excitatory neurotransmitter glutamate (Bryant et al., 2019). Magnetic resonance imaging for measuring regional cerebral blood flow has shown changes in interregional connectivity patterns of the brain in response to ketamine application affecting centers such as the prefrontal and cingulate cortex and the thalamus (Bryant et al., 2019) which contribute to the control of states of consciousness (e.g., Dehaene and Changeux, 2011). Therefore, clinical signs of NDE-like perceptions following intake of subanesthetic doses of ketamine (Jansen, 1996) may be related to changes in functional connectivity in the brain leading to changes in consciousness. In fact, the five most common category themes in the narratives of people, who had taken ketamine, were the same as the five most common themes found in the NDE reports (Martial et al., 2019). Another serotonergic drug, DMT (N,N-dimethyltryptamine), may also cause NDE-like phenomena. The controlled administration of DMT to volunteers led to very similar expressions of 15 of the 16 items of the Greyson (1983) scale compared to persons who had experienced NDEs (Timmermann et al., 2018). These similarities suggest a common origin of NDEs and the various NDE themes in the ASCs that can be induced *via* drug consumption (Earleywine, 2010; Souza and Perry, 2018; Martial et al., 2019) in the brains of consumers. Therefore, great care has to be taken in order to consider the ethical background when planning the controlled application of drugs in experimental conditions of measurement of brain activity for testing possible NDE-like occurrence associated with ASCs.

Interestingly, the *N*-methyl-D-aspartate receptor (NMDAR) antagonist ketamine and DMT, an endogenous agonist of sigma-1 receptors, are known to inhibit spreading depolarization (Fontanilla et al., 2009; Reinhart and Shuttleworth, 2018; Santos et al., 2019; Szabó et al., 2021). Hypothetically, their ability to elicit NDE-like phenomena may suggest that an endogenous NMDAR antagonist and/or DMT or other sigma-1 agonists are released in life-threatening situations prior to brain-toxic spreading depolarization, and thereby delaying the onset of spreading depolarization and/or reducing its toxicity. Such possible neuroprotective effects of drugs should further be investigated.

### Anesthesia

General anesthesia is supposed to provide a level of unconsciousness necessary for the performance of major surgery. Observations have shown that some people can remember and report about experiences of conscious awareness, even when they were supposedly unconscious under anesthesia (Andrade and Deeprose, 2007; Bell, 2012; Woerlee, 2013). Insufficient levels of anesthesia combined with the application of muscle relaxants seem to be one of the main causes of preserved or modified awareness in patients during surgery (Ghoneim, 2000; Osterman et al., 2001). Cobcroft and Forsdick (1993) analyzed letters of 187 patients from Australia and New Zealand who experienced details of their environment and/or the handling of their body during surgery under general anesthesia. Among these experiences were NDE-like phenomena such as OBEs, moving in a tunnel, seeing operation details and bright light, and hearing conversations among surgeons.

### Epileptic seizures

Many features of NDEs have been described as symptoms of epileptic seizures with the focus on the temporal lobe of the cerebral cortex (Britton and Bootzin, 2004) or, in case of ecstatic epilepsy, involving the anterior insula (Picard et al., 2021). Changes in activation of the temporal lobe were found when persons had mystical or religious experiences (van Elk and Aleman, 2017; Peinkhofer et al., 2019). More persons who had NDEs with transcendental aspects during life-threatening events were found to have temporal lobe paroxysmal EEG activity than control subjects (21.7 vs. 5%; Britton and Bootzin, 2004). Further, the incidence of epileptiform activity found in the Britton and Bootzin (2004) NDE sample exceeded the incidence found in normal, non-clinical populations (0.4%) and in non-epileptic clinical populations (2–3%; Binnie and Stefan, 1999). Consequently, these results suggested that some altered temporal lobe functions may be involved in generating NDE themes.

### Electrical stimulations of the brain

**FIGURE 1:**
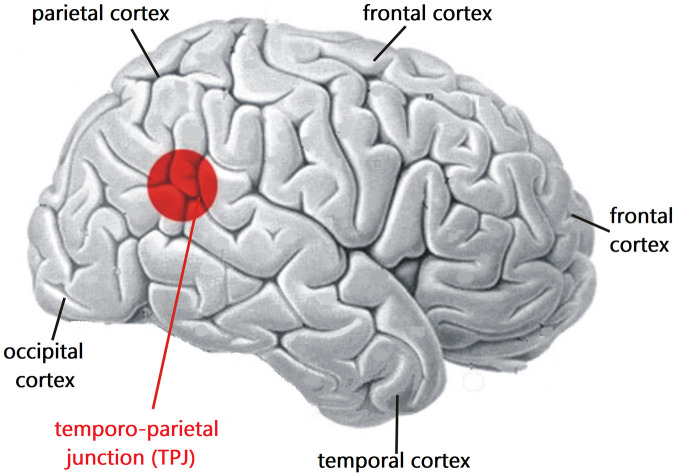
Location of the temporo-parietal junction (TPJ) on the right-hemisphere cerebral cortex. The TPJ is located where the temporal and parietal lobes meet, not far from the occipital (visual) cortex. Studies have shown that functional modifications of the activation of the TPJ can lead to generating OBEs (compare text). The basic brain model has been modified from Nieuwenhuys et al. (1980), their Fig. 7 (copyright permission by Springer Nature).

Penfield (1955), a pioneer of functional mapping of the human neocortex *via* local electrical stimulation, found that some fully conscious subjects stimulated in the temporal lobe or the temporo-parietal junction (TPJ) reported an altered sense of time and past visual images reminding of some reported experiences from NDErs. More recently, Blanke and Dieguez (2009) observed many features and themes of NDEs including OBEs in reports of persons after electrical stimulation of limbic brain regions such as the hippocampus, amygdala, parahippocampal gyrus, and neocortical temporal areas including the TPJ (compare Figure 1). A whole spectrum of NDE themes was generated by brain stimulation during the course of electroconvulsive therapy to treat a depressive, suicidal patient (Floyd, 1996).

### Modifications of the supply of blood gases to the brain (hypoxia, anoxia, hypercarbia)

A reduction of the blood flow to the brain generates cerebral hypoxia or, in more dramatic cases, anoxia causing LOC and the occurrence of NDE phenomena (Lempert et al., 1994; van Lommel, 2010). A study on 52 cardiac arrest survivors has demonstrated a higher concentration of carbon dioxide and potassium levels in the arterial blood in patients who had had an NDE compared to those who had not (Klemenc-Ketis et al., 2010). They suggested that higher carbon dioxide levels may have an excitatory effect on the limbic system, which might result in NDE-like experiences (Petersson Bouin, 2000). The possible relation between the limbic system activation and NDE-like experiences has also been reported by Blanke and Dieguez (2009).

Cerebral hypoxia could also induce feelings of happiness and euphoria and of having minds clearer than normal, and intellects keener than before (van Liere and Stickney, 1963; Cudaback, 1984). Such observations correspond well to reports of NDErs sounding like “vivid memory, veridical, more real than real, realer than real, super real” (Schlieter, 2018). This psychophysiological experience is an illusion as demonstrated by experimental studies of cerebral hypoxia with English fighter pilots during World War 2 (Woerlee, 2013).

### In summary

Our glimpse on several generally known and well-studied classes of modifications of brain function revealed common effects of the modifications on the perception of the affected persons. The reported perceptual experiences were often similar to if not identical with those of NDE reports and, together, covered the NDE themes of the Greyson (1983) scale. Different from original NDEs, the NDE-like phenomena from the mentioned classes of modification of brain functions may be repeated with scientific scrutiny and, thus, be validated. In this sense, we mentioned modifications of brain functions as adequate models for explaining NDEs. And we will add another so far largely unvalued model in the NDE context because the original data have been recorded in the context of testing the physical robustness of fighter pilots.

## Approach *via* modification of brain functions of fighter pilots under gravitational stress

During aerial combats, fighter pilots are exposed to high head-to-foot acceleration (+ Gz stress; Davis et al., 2008). As result, blood is pooled in the abdomen and in the extremities generating reduced blood flow to the head with cerebral hypotension and cephalic nervous system ischemia. In order to understand the conditions of + Gz-induced LOC (G-LOC, Whinnery, 1991), experiments with pilots accelerated in ground-based centrifuges have been conducted. In these experiments, many physiological and psychological parameters of the video-recorded and physiologically controlled pilots have been evaluated in relation to the physical parameters of the + Gz stress (Whinnery and Whinnery, 1990; Whinnery, 1991). In the course of the studies, initially 500 states of acceleration to unconsciousness have been evaluated within 11 years. A later study listed nearly 1000 centrifuge G-LOC episodes recorded and analyzed during 16 years (Whinnery, 1997). Kinetics of becoming unconscious, staying unconscious for a certain time, and becoming conscious again could be related to the behavior of neuronal populations under anoxic stress as observed in humans and animals (Dreier et al., 2018, 2022; Schramm et al., 2020; Lemale et al., 2022). This could well correspond to neuronal physiologic and brain functional phenomena of anoxia (Woerlee, 2013). Thus, a decrease of blood flow to the head could be associated with the length of unconsciousness depending on the magnitude of the reduced blood flow as shown in experiments on animals (Werchan et al., 1996).

The lengths of the unconsciousness episodes averaged 12 s with a range of 2 to 38 s. The average time of altered blood flow to the central nervous system causing loss and recovery of consciousness was estimated near 15–20 s (Whinnery, 1997). In many episodes, the unconscious pilots experienced NDE-like perceptions such as tunnel visions, seeing bright light, a peaceful sense of floating, brief observations of images from the past, and pictures of living persons. Increased periods of unconsciousness led to so-called memorable dreams (Whinnery and Whinnery, 1990), actually dreamlets of short duration that met, however, the characteristic features of dreams (Hobson, 1988; Fagioli, 2002) such as emotional intensity, detailed sensory imagery, illogical content, and inorganized, uncritical acceptance, and sometimes difficulty in remembering once it was over. In addition, dream characteristics such as myoclonic convulsions and automatic movements were incorporated in the dreamlets that could mimic some aspects of autoscopy (Whinnery and Jones, 1987; Whinnery and Whinnery, 1990; Whinnery, 1997). Together, it seems that the pilots could experience both NDE-like perceptions and fractions of regular dreams during the period of their unconsciousness.

Whinnery et al. (2014) provided a rather complete description of the kinetics and characteristics of loss and recovery of consciousness in the G-LOC tests due to the response of the brain to transient alterations of the normal blood supply. The G-LOC tests revealed further interesting observations in relation to NDEs: OBEs and autoscopy have been recorded mainly in longer-duration episodes or multiple, closely spaced G-LOCs, as many as five within a 15-min period (Whinnery and Jones, 1987; Whinnery, 1997). This may indicate that OBEs are likely associated with increasingly severe ischemic insults to the brain. Observations from dreams and myoclonic convulsions occurring prior to the end of unconsciousness and from the return to consciousness suggested, with regard to memory formation and recall, the following: The point of last memory generally preceded the LOC, indicating that memory was compromised prior to LOC. On the opposite, memory processes returned prior to the recovery of full consciousness (Whinnery, 1997; Whinnery and Forster, 2013; Whinnery et al., 2014). These observations could well be applied to NDEs and could resolve several questions regarding what NDErs could have known and reported about their situation during the unconscious phase. They support the above-mentioned suggestion derived from the recovery of the hypoxic rat brain (Schramm et al., 2020) that the themes of NDEs may be generated rather during the phase of recovery from unconsciousness than in the initial phase of becoming unconscious.

### In summary

What do the G-LOC tests contribute to the understanding of the neuro-functional bases of NDEs including OBEs? The unarguable strength of the results of the G-LOC studies is their proof that:

– a temporarily reduced blood flow to the brain causes unconsciousness giving rise to a number of extraordinary perceptions which can occur in rapid successions within a few seconds of unconsciousness and in the transition from the conscious to the unconscious phase and/or back to the conscious phase. This is strong support of the results from the clinical observations and recordings with modified air supply (hypoxia, anoxia) to the brain as mentioned before.
– the generation of OBEs compared to other NDE themes appears as a response to increased ischemic stress.

Major perceptions and experiences reported after G-LOC episodes are shared with NDEs. They include tunnel vision and bright lights, floating sensations, automatic movement, autoscopy, OBEs, not wanting to be disturbed, pleasurable sensations, psychological alterations of euphoria and dissociation, the inclusion of friends and family, the inclusion of prior memories and thoughts, and a strong urge to understand the experience. According to Greyson’s (1983) scale, certainly many G-LOC episodes would have been evaluated as NDEs.

## Explanatory models of near-death experiences/out-of-body experiences: Toward the ultimate origins of the phenomena

Approaches in controlled medical and/or experimental settings–*via* the use of drugs and anesthetics, recordings during epileptic seizures, electrical brain stimulation and modifications of blood flow to the brain including G-LOC tests—have all been successful to generate perceptual experiences that mimic NDEs. Further, lucid dreaming and hallucinations of various genesis can reproduce NDE themes (e.g., Slade and Bentall, 1988; Britton and Bootzin, 2004; Nelson et al., 2007; Allen et al., 2008; Blackmore, 2012; Nelson, 2014; Shermer, 2018). The common expression of these observations are ASCs (Charland-Verville et al., 2014, 2020) pointing toward neurological origins (e.g., Blanke, 2004; Blanke and Dieguez, 2009). The question is, which brain functions go awry during traumatic or other events leading to NDEs, NDE-like phenomena or experiences under G-LOC (G-LOCEs)?

Near-death experiences and G-LOCEs are recalls of some past experiences. Therefore, parts of the temporal lobe and the limbic system which are involved in generating declarative (episodic, semantic) and emotional memory (e.g., LaBar and Cabeza, 2006; Vignal et al., 2007; Greenberg et al., 2009; Dolcos et al., 2017; Cooper and Ritchey, 2019) can be expected to be involved in producing NDEs and G-LOCEs. In fact, various experiences have been reported after memory activation by electrical stimulation or during epileptic seizures, and in cases of brain disorders and pathologies. These experiences concern auditory, visual, and religious perceptions (Persinger, 1983; Lezak et al., 2012), mystical perceptions (Britton and Bootzin, 2004; Marsh, 2010), rapid life reviews (Devinsky et al., 1989), events of pleasure, ecstasy, and euphoria (Vuilleumier et al., 1997), meeting deceased relatives or friends (Ionâşescu, 1960), experiencing presences (Persinger, 1983; Cristofori et al., 2016), vivid visual and auditory hallucinations, sudden insights, ego-alien intrusions, mystical encounters and intense personal meanings to the experience reported (Gloor et al., 1982; Thalbourne et al., 2003; Cristofori et al., 2016). These themes largely overlap with the NDE and G-LOCE themes providing sufficient evidence that areas of the temporal cortex and the limbic system may contribute to generating the major content of NDE and G-LOC themes.

Positioned at the transition from the temporal to the parietal and occipital cortices, the (TPJ; Figure 1) plays an important role in the comprehension of our own body (self-consciousness of the body) and its situation in space (e.g., Arzy et al., 2006; Ionta et al., 2011; Guterstam et al., 2015; Eddy, 2016). The involvement of the TPJ in generating OBEs has been shown by electrical stimulation mainly of the right hemisphere TPJ which could elicit OBEs (Blanke et al., 2002, 2004, 2005; De Ridder et al., 2007; Blanke and Metzinger, 2009; Yong, 2011). One line of reasoning is that disintegration of multisensory processing in the TPJ, for example by electrical stimulation, leads to the disruption of several phenomenological and cognitive aspects of self-processing, causing illusory reduplication, illusory self-location, illusory perspective and illusory acting that are either experienced as OBEs or labeled as belonging to the class of OBEs (Blanke et al., 2004, 2005; Hirsch, 2005; De Ridder et al., 2007; Aspell and Blanke, 2009; Vanhaudenhuyse et al., 2009; Heydrich et al., 2010; Aglioti and Candidi, 2011; Ionta et al., 2011). Similarly, new techniques of creating virtual realities can experimentally dissociate the perception of the self from that of the own physical body. In other words, self-consciousness can be attributed to an illusory body part or even to an avatar (Ehrsson et al., 2005; Sanchez-Vives and Slater, 2005; Ehrsson, 2007; Lenggenhager et al., 2007; Yong, 2011). This happens, for example, when conflicting information from the eyes and the sense of touch or the vestibular sense (body position in space) has to be processed in the TPJ (Lenggenhager et al., 2007). The result of the misalignment of sensory information in the TPJ can lead to illusory body perceptions including OBEs.

Since the TPJ is part of a neural network defining the bodily self together with the parietal cortex, parts of the cingulate cortex, and parts of the prefrontal cortex (Vogeley and Fink, 2003; Blanke and Metzinger, 2009; Blanke, 2012; Guterstam et al., 2015; Blondiaux et al., 2021) alterations of TPJ activity in the course of NDE, NDE-like and G-LOCE generation are expected to contribute to NDE and G-LOC themes explicitly referring to the self, as reported as “I” in a first-person perspective. Similarly, NDE and G-LOCE themes referring to visual phenomena (seeing a bright light, a dark tunnel, etc.) may depend on altered activity in the occipital cortex, where the primary visual areas are located (e.g., Wandell et al., 2007). In perspective, further studies are promising to show that certain NDE themes are coupled with a focus of change of the neural activity in certain brain areas associated with processing the content of the respective themes. Such attempts in neurophenomenological localization *via* EEG recordings started recently (Martial et al., 2019). Further EEG studies or other recordings can also test the hypothesis based on a synthesis of data from the studies of Thonnard et al. (2013) and Palmieri et al. (2014), namely that NDE memories are hallucination-like memories of actually perceived hallucinations.

## General conclusion

Neuroactive substances such as anesthetics and other drugs, epileptic seizures and brain stimulation and, especially, ischemic stress (hypoxia, anoxia) of the brain, all these modifications of brain functioning have been shown to generate subjective perceptions closely covering those of NDEs including OBEs. Therefore, neuro-functional models of NDE including OBE generation offer scientifically appropriate causal explanations for the occurrence of NDEs, possibly as hallucinations of a brain in ASCs. Such alterations are very likely expressions of physiological changes in a brain under ischemic stress as has been shown mainly after cardiac arrest in persons attested to be clinically dead. Neuro-functional NDE models may be used in further studies under well-controlled clinical/laboratory settings to gain more insight into NDE-specific questions such as the generation of specific NDE themes by local cortical activity, or which actual state of consciousness may allow NDE memory content to be saved. In general, the study of NDE models will contribute to the understanding of how brain activity can generate and represent subjective experiences at all. NDEs actually seem to be promising gates to the study of brain functions, especially in situations in which states of altered consciousness may be traced back to defined measures or sets of measures of brain activity.

## Data availability statement

The original contributions presented in this study are included in the article/Supplementary material, further inquiries can be directed to the corresponding authors.

## Author contributions

RR initiated the project, supplied the relevant literature, and wrote the first draft. GE contributed ideas and literature, deepened the scientific background, and wrote the final manuscript together with RR. Both authors contributed to the article and approved the submitted version.
