# Supplementary material for: Neuro-functional modeling of near-death experiences in contexts of altered states of consciousness
Source: Front Psychol. 2023 Jan 18;13:846159. doi: 10.3389/fpsyg.2022.846159 (PMC9891231; doi:10.3389/fpsyg.2022.846159)
Supplement: Supplementary file 1 [file Table_1.DOCX]

**Supplement 1: Example story of a car accident leading to a report of near-death experience.**

George, after a day of work, was on his way back home by car. At that time of the day, the traffic was heavy and the road was wet. He was concentrated on his driving. Suddenly, a car turned off to the right in front of him forcing him to brake heavily, the car behind him bumped his car forcing him to deviate from the road and fall down a slope. After the shock, he lost consciousness. Being conscious again, he found himself in a hospital bed connected to bottles and instruments via several lines. An emergency physician came asking about his feeling and about what he remembered from his crash. Surprisingly, after having complained about his left shoulder and the numerous tubes restricting his movements, George was quick and eager to tell a strange story. After a black screen, he told he was flying like a rocket toward a light spot in space. He traveled at a high speed toward the spot which became bigger and bigger and more brilliant. During this cosmic travel, he perceived, like in a slide show, aspects of his past life. When getting in a space full of light, he spotted beings of light, some of them seemed to be known to him. In these surroundings, he felt completely loved and accepted. He was able to communicate telepathically with one of the beings who described the place as residing out of time with all the knowledge of the universe being present. Simultaneously he experienced ineffable feelings of love and peace.
