# Supplementary material for: Neuro-functional modeling of near-death experiences in contexts of altered states of consciousness
Source: Front Psychol. 2023 Jan 18;13:846159. doi: 10.3389/fpsyg.2022.846159 (PMC9891231; doi:10.3389/fpsyg.2022.846159)
Supplement: Supplementary file 2 [file Table_2.docx]

**Supplement 2: NDE themes as listed in Table 2 (Greyson, 1983, final NDE scale) as *component and question*.**

Cognitive

1. Did time seem to speed up?
2. Were your thoughts speeded up?
3. Did scenes from your past come back to you?
4. Did you suddenly seem to understand everything?

Affective

1. Did you have a feeling of peace or pleasantness?
2. Did you have a feeling of joy?
3. Did you feel a sense of harmony or unity with the universe?
4. Did you see of feel surrounded by a brilliant light?

Paranormal

1. Were your senses more vivid than usual?
2. Did you seem to be aware of things going on elsewhere as if by ESP? [our addition: ESP means extrasensory perception]
3. Did scenes from the future come to you?
4. Did you feel separated from your physical body?

Transcendental

1. Did you seem to enter some other, unearthly world?
2. Did you seem to encounter a mystical being or presence?
3. Did you see deceased spirits or religious figures?
4. Did you come to a border or point of no return?
